# Supplementary material for: Mexican Strains of Anaplasma marginale: A First Comparative Genomics and Phylogeographic Analysis
Source: Pathogens. 2022 Aug 2;11(8):873. doi: 10.3390/pathogens11080873 (PMC9415054; doi:10.3390/pathogens11080873)
Supplement: Supplementary file 1 [file pathogens-11-00873-s001.zip › pathogens-1809943-Figure_S1.pdf]

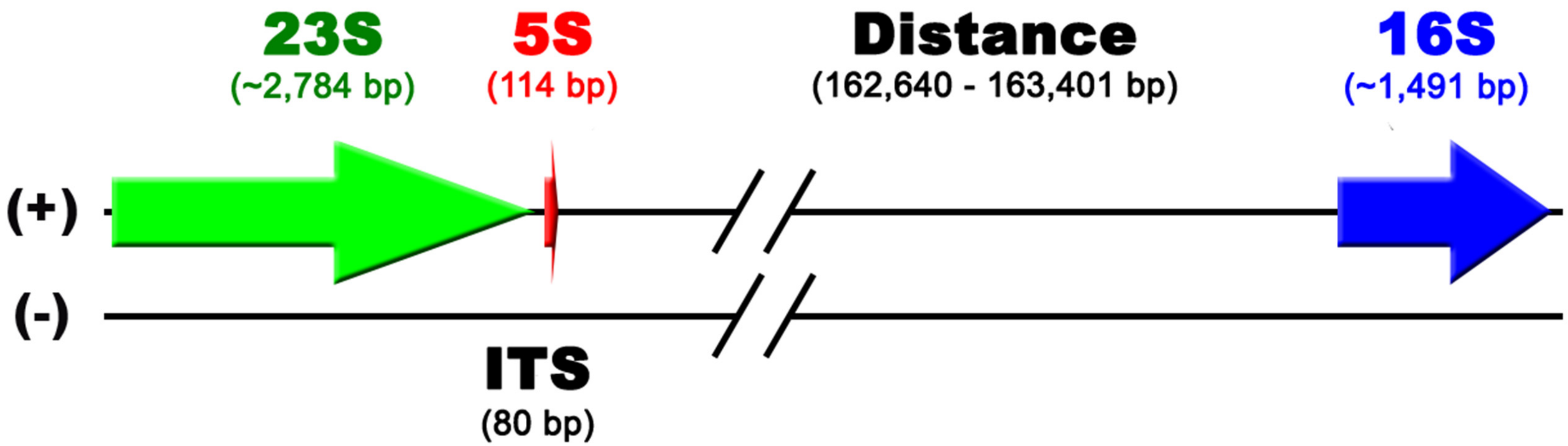

Figure S1. Mapping of the rRNA genes (arrows) of *Anaplasma marginale* genomes. The two complete genomes (strains Florida and St. Maries) and the four closed genomes assembled in a single incomplete chromosome (strains Dawn, Gypsy Plains, Jaboticabal and Palmeira) contain a distance from 162,640 to 163,401 bp between the 5S and 16S rRNA genes. The 18 draft genomes (including the seven Mexican strains) contain the 23S-ITS-5S and 16S rRNA genes located in different contigs.
