# Supplementary material for: Mexican Strains of Anaplasma marginale: A First Comparative Genomics and Phylogeographic Analysis
Source: Pathogens. 2022 Aug 2;11(8):873. doi: 10.3390/pathogens11080873 (PMC9415054; doi:10.3390/pathogens11080873)
Supplement: Supplementary file 1 [file pathogens-11-00873-s001.zip › pathogens-1809943-Figure_S2.pdf]

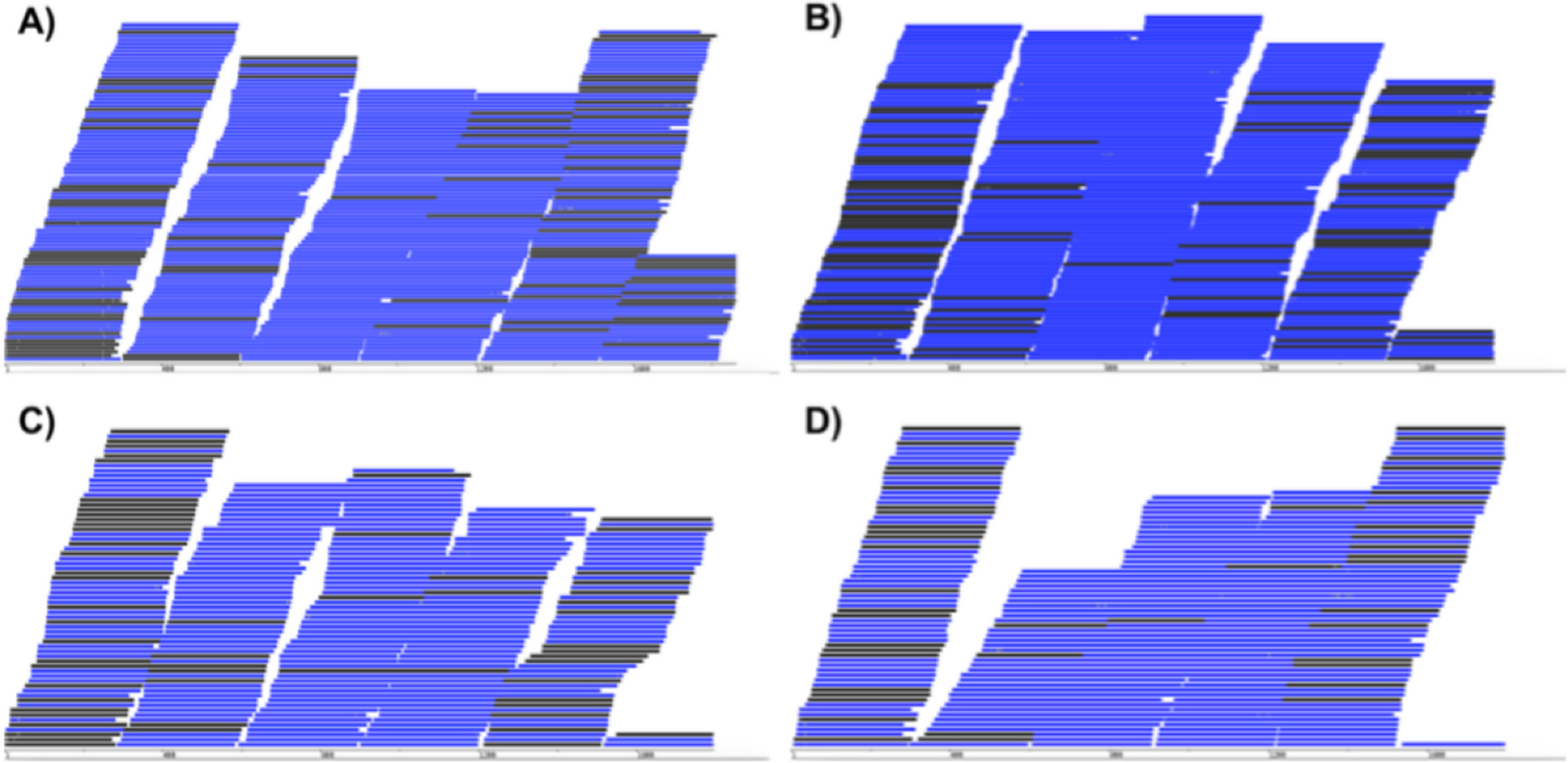

Figure S2. Representative diagram showing the result of the alignment between reads obtained from the genome sequencing (blue and black lines with 300 bases of length) and *msp1a* genes (white line at the bottom with ~1.8 Mb of length) of the Mexican strains MEX-15-099-01 from Texcoco, Estado de México (A); MEX-17-017-01 from Puente de Ixtla, Morelos (B); MEX-30-184-02 from Tlapacoyan, Veracruz (C); and MEX-31-096-01 from Tizimín, Yucatán (D). Paired reads are shown in blue and unpaired reads are shown in black. The repeats structure of the *msp1a* genes is located on the left side.
