# Supplementary material for: Mexican Strains of Anaplasma marginale: A First Comparative Genomics and Phylogeographic Analysis
Source: Pathogens. 2022 Aug 2;11(8):873. doi: 10.3390/pathogens11080873 (PMC9415054; doi:10.3390/pathogens11080873)
Supplement: Supplementary file 1 [file pathogens-11-00873-s001.zip › pathogens-1809943-Table_S1.pdf]

Table S1. 24 genomes of *Anaplasma marginale* strains reported to date in the GenBank database.

Organism	Country	Accession Number
A. marginale Dawn	Australia	CP006847.1
A. marginale Gypsy Plains	Australia	CP006846.1
A. marginale Jaboticabal	Brazil	CP023731.1
A. marginale Palmeira	Brazil	CP023730.1
A. marginale MEX-01-001-01	Mexico	QLIV00000000.1
A. marginale MEX-14-010-01	Mexico	VTSO00000000.1
A. marginale MEX-15-099-01	Mexico	VTWW00000000.1
A. marginale MEX-17-017-01	Mexico	VTCX00000000.1
A. marginale MEX-30-184-02	Mexico	VTCY00000000.1
A. marginale MEX-30-193-01	Mexico	VTCZ00000000.1
A. marginale MEX-31-096-01	Mexico	VTWV00000000.1
A. marginale Puerto Rico	Puerto Rico	ABOQ00000000.1
A. marginale Florida	United States	CP001079.1
A. marginale Florida	United States	AFMS00000000.1
A. marginale Florida Relapse	United States	AFMT00000000.1
A. marginale Mississippi	United States	ABOP00000000.1
A. marginale Okeechobee	United States	AFMV00000000.1
A. marginale Oklahoma	United States	AFMX00000000.1
A. marginale Oklahoma-2	United States	PKOF00000000.1
A. marginale South Idaho	United States	AFMY00000000.1
A. marginale St. Maries	United States	CP000030.1
A. marginale St. Maries	United States	AFMU00000000.1
A. marginale Virginia	United States	ABOR00000000.1
A. marginale Washington Okanogan	United States	AFMW00000000.1
