# Supplementary material for: Mexican Strains of Anaplasma marginale: A First Comparative Genomics and Phylogeographic Analysis
Source: Pathogens. 2022 Aug 2;11(8):873. doi: 10.3390/pathogens11080873 (PMC9415054; doi:10.3390/pathogens11080873)
Supplement: Supplementary file 1 [file pathogens-11-00873-s001.zip › pathogens-1809943-Table_S2.pdf]

**Table S2.** Nucleotide content of four genomes assembled in a single incomplete chromosome of *Anaplasma marginale*.

| Symbol | Meaning | *A. marginale* Dawn | *A. marginale* Gypsy Plains | *A. marginale* Jaboticabal | *A. marginale* Palmeira |
|---|---|---|---|---|---|
| G | G | 272,774 | 281,479 | 294,006 | 293,694 |
| A | A | 279,171 | 288,214 | 300,583 | 300,407 |
| T | T | 278,699 | 287,644 | 299,269 | 299,243 |
| C | C | 279,147 | 288,040 | 300,275 | 300,063 |
| R | G / A | 73 | 47 | 195 | 302 |
| Y | T / C | 79 | 71 | 240 | 346 |
| M | A / C | 49 | 45 | 127 | 220 |
| K | G / T | 43 | 45 | 130 | 224 |
| S | G / C | 43 | 48 | 138 | 181 |
| W | A / T | 42 | 47 | 113 | 179 |
| H | A / T / C | 1 | 3 | 1 | 0 |
| B | G / T / C | 4 | 3 | 1 | 2 |
| V | G / A / C | 2 | 2 | 0 | 3 |
| D | G / A / T | 1 | 2 | 0 | 4 |
| N | A / T / G / C | 86,632 | 52,932 | 243 | 332 |
| **G+A+T+C (%)** | | 1,109,791 (92.73%) | 1,145,377 (95.56%) | 1,194,133 (99.90%) | 1,193,407 (99.85%) |
| **R+Y+M+K+S+W+H+B+V+D+N (%)** | | 86,969 (7.27%) | 53,245 (4.44%) | 1,188 (0.10%) | 1,793 (0.15%) |
| **Total length (bp)** | | 1,196,760 | 1,198,622 | 1,195,321 | 1,195,200 |
