# Supplementary material for: Mexican Strains of Anaplasma marginale: A First Comparative Genomics and Phylogeographic Analysis
Source: Pathogens. 2022 Aug 2;11(8):873. doi: 10.3390/pathogens11080873 (PMC9415054; doi:10.3390/pathogens11080873)
Supplement: Supplementary file 1 [file pathogens-11-00873-s001.zip › pathogens-1809943-Table_S3.pdf]

Table S3. Features of the rRNA genes in the 24 genomes of *Anaplasma marginale*.

Organism	23S rRNA		ITS length (bp)	5S rRNA		Distance (bp)	16S rRNA	
	Contig	Length (bp)		Contig	Length (bp)		Contig	Length (bp)
A. marginale Dawn	Chr	2,784	80	Chr	114	163,391	Chr	1,491
A. marginale Gypsy Plains	Chr	2,784	80	Chr	114	163,401	Chr	1,491
A. marginale Jaboticabal	Chr	2,784	80	Chr	114	162,640	Chr	1,491
A. marginale Palmeira	Chr	2,784	80	Chr	114	162,670	Chr	1,491
A. marginale MEX-01-001-01	6	2,784	80	6	114	NA	8	1,491
A. marginale MEX-14-010-01	12	2,784	80	12	114	NA	1	1,491
A. marginale MEX-15-099-01	12	2,784	80	12	114	NA	7	1,491
A. marginale MEX-17-017-01	10	2,784	80	10	114	NA	7	1,491
A. marginale MEX-30-184-02	17	2,784	80	17	114	NA	8	1,491
A. marginale MEX-30-193-01	16	2,784	80	16	114	NA	7	1,491
A. marginale MEX-31-096-01	16	2,784	80	16	114	NA	4	1,491
A. marginale Puerto Rico	14	2,783	80	14	114	NA	25	1,491
A. marginale Florida	Chr	2,785	80	Chr	114	162,752	Chr	1,491
A. marginale Florida	183	2,784	80	183	114	NA	21	1,376
A. marginale Florida Relapse	24	2,784	80	24	114	NA	88	1,491
A. marginale Mississippi	27	2,782	80	27	114	NA	44	1,491
A. marginale Okeechobee	556	2,784	80	556	114	NA	561	1,491
A. marginale Oklahoma	98	2,784	80	98	114	NA	73	1,491
A. marginale Oklahoma-2	15	2,784	80	15	114	NA	1	1,491
A. marginale South Idaho	50	2,784	80	50	114	NA	56	1,491
A. marginale St. Maries	Chr	2,784	80	Chr	114	163,334	Chr	1,491
A. marginale St. Maries	75	2,784	80	75	114	NA	77	1,491
A. marginale Virginia	19	2,782	80	19	114	NA	30	1,491
A. marginale Washington Okanogan	294	2,783	80	294	114	NA	299	1,491

Chr: Chromosome

NA: Not Available
