# Supplementary material for: Mexican Strains of Anaplasma marginale: A First Comparative Genomics and Phylogeographic Analysis
Source: Pathogens. 2022 Aug 2;11(8):873. doi: 10.3390/pathogens11080873 (PMC9415054; doi:10.3390/pathogens11080873)
Supplement: Supplementary file 1 [file pathogens-11-00873-s001.zip › pathogens-1809943-Table_S4.pdf]

Table S4. The tRNA codon frequency of the 24 genomes of *Anaplasma marginale*.

Codon	Amino Acid	19 genomes*	Strains Dawn (incomplete chromosome) and Florida (draft genome)	Strain Okeechobee (draft genome)	Strain South Idaho (draft genome)	Strain Washington Okanogan (draft genome)
TTT	Phe					
TTC	Phe	1	1	1	1	2
TTA	Leu	1	1	1	1	1
TTG	Leu	1	1	1	1	1
CTT	Leu					
CTC	Leu	1	1	1	1	1
CTA	Leu	1	1	1	2	1
CTG	Leu	1	1	1	1	1
ATT	Ile					
ATC	Ile	1	1	1	2	2
ATA	Ile					1
ATG	Met	3	3	3	3	4
GTT	Val					
GTC	Val	1	1	1	1	1
GTA	Val	1	1	1	2	1
GTG	Val					
TCT	Ser					
TCC	Ser	1	1	1	1	1
TCA	Ser	1	1	1	1	2
TCG	Ser					
CCT	Pro					
CCC	Pro	1	1	1	1	1
CCA	Pro	1	1	1	1	1
CCG	Pro					
ACT	Thr					
ACC	Thr	1	1	1	1	2

ACA	Thr	1	1	1	2	1
ACG	Thr					
GCT	Ala					
GCC	Ala					
GCA	Ala	1	1	1	2	2
GCG	Ala					
TAT	Tyr					

Table S4. The tRNA codon frequency of the 24 genomes of *Anaplasma marginale* (continuation).

**Codon**	**Amino Acid**	**19 genomes***	**Strains Dawn (incomplete chromosome) and Florida (draft genome)**	**Strain Okeechobee (draft genome)**	**Strain South Idaho (draft genome)**	**Strain Washington Okanogan (draft genome)**
TAC	Tyr	1	1	1	1	2
TAA	Stop					
TAG	Pyl					
CAT	His					
CAC	His	1	1	1	2	2
CAA	Gln	1	1	1	1	2
CAG	Gln					
AAT	Asn					
AAC	Asn	1	1	1	1	1
AAA	Lys	1	1	1	1	2
AAG	Lys	1	1	1	1	1
GAT	Asp					
GAC	Asp	1	1	1	1	2
GAA	Glu	1	1	1	1	1
GAG	Glu					
TGT	Cys					
TGC	Cys	1	1	1	2	1
TGA	SeC					
TGG	Trp	1	1	1	2	2

CGT	Arg	1	1	1	1	1
CGC	Arg					
CGA	Arg			1	1	1
CGG	Arg	1	1	1	1	1
AGT	Ser					
AGC	Ser	1	1	1	1	2
AGA	Arg	1	1	1	1	1
AGG	Arg	1	1	1	1	1
GGT	Gly					
GGC	Gly	1		1	2	1
GGA	Gly	1	1	1	1	1
GGG	Gly	1		1	1	1
**Number of tRNA genes**		**37**	**35**	**38**	**47**	**52**

*Including the strains: Gypsy Plains, Jaboticabal, Palmeira, MEX-01-001-01, MEX-14-010-01, MEX-15-099-01, MEX-17-017-01, MEX-30-184-02, MEX-30-193-01, MEX-31-096-01, Puerto Rico, Florida (complete genome), Florida Relapse, Mississippi, Oklahoma, Oklahoma-2, St. Maries (complete and draft genomes) and Virginia.
