# Supplementary material for: Mexican Strains of Anaplasma marginale: A First Comparative Genomics and Phylogeographic Analysis
Source: Pathogens. 2022 Aug 2;11(8):873. doi: 10.3390/pathogens11080873 (PMC9415054; doi:10.3390/pathogens11080873)
Supplement: Supplementary file 1 [file pathogens-11-00873-s001.zip › pathogens-1809943-Table_S5.pdf]

Table S5. Features of *msp1a* genes in the seven draft genomes of Mexican strains of *Anaplasma marginale*.

Organism	Contig	Chain	Initial position	Final position	Length (bp)	Length (aa)
A. marginale MEX-01-001-01	1	-	36,513	34,480	2,034	678
A. marginale MEX-14-010-01	1	-	36,147	34,201	1,947	649
A. marginale MEX-15-099-01	1	+	214,493	216,346	1,854	618
A. marginale MEX-17-017-01	1	-	36,095	34,311	1,785	595
A. marginale MEX-30-184-02	1	-	39,711	37,927	1,785	595
A. marginale MEX-30-193-01	1	+	215,085	217,025	1,941	647
A. marginale MEX-31-096-01	1	-	39,711	37,927	1,785	595
