# Supplementary material for: Mexican Strains of Anaplasma marginale: A First Comparative Genomics and Phylogeographic Analysis
Source: Pathogens. 2022 Aug 2;11(8):873. doi: 10.3390/pathogens11080873 (PMC9415054; doi:10.3390/pathogens11080873)
Supplement: Supplementary file 1 [file pathogens-11-00873-s001.zip › pathogens-1809943-Table_S6.pdf]

Table S6. MSP1a RI and RL repeat sequences associated to four ecoregions reported by Estrada-Peña et al. [19].

MSP1a repeat	Unique sequences	Other sequences present in strains recorded from more than one ecoregion
RI / Ecoregion 1	4, 8, 16, 56, 60, 64, 67, Γ, π, τ	A, B, D, T, 13, 23, α
RI / Ecoregion 2	28, 48, 53, E, F, ε	A, B, L, T, 13, 23, α
RI / Ecoregion 3	1, 3, 5, 6, 27, 33, 34, 39, M, O, Q, U	A, D
RI / Ecoregion 4	I, J, K	A, B, L, α
RL / Ecoregion 1	8, 9, 12, 15, 59, 61, 66	B, C, M, 18, 27, Γ
RL / Ecoregion 2	10, 31, 52, π, β	F, H, M, 27, Γ
RL / Ecoregion 3	3, 7, 35, 37, 38, 44, E, N, P, Q, U, ρ	B, F, H, 18, Γ
RL / Ecoregion 4	none	B, C, H
