# Supplementary material for: Mexican Strains of Anaplasma marginale: A First Comparative Genomics and Phylogeographic Analysis
Source: Pathogens. 2022 Aug 2;11(8):873. doi: 10.3390/pathogens11080873 (PMC9415054; doi:10.3390/pathogens11080873)
Supplement: Supplementary file 1 [file pathogens-11-00873-s001.zip › pathogens-1809943-Table_S7.pdf]

Table S7. General features of the four ecoregions reported by Estrada-Peña et al. [19].

	ECOREGIONS			
	**1**	**2**	**3**	**4**
**Main geographical regions worldwide**	Large areas of central Africa and central South America, primarily Argentina and southern Brazil.	Vast areas of the Mesoamerican corridor, northern South America and a small territory of eastern South Africa.	Central South Africa and scattered parts of southern USA and Mexico.	Large areas of USA.
**NDVI* values**	Medium to high NDVI values with a clear seasonal decrease between June and September.	High NDVI along the year without seasonal variability.	Lowest NDVI values and little change across the year.	A clear NDVI signature, very low between November and March and then rising to reach maximum levels around July.
**Temperature values**	The highest recorded temperature.	Similar to those in ecoregion 1.	Lower temperature values.	The coldest among all the ecoregions.
**Rainfall values**	Around 1,000 mm/year.	Around 1,500 mm/year.	Minimum rainfall.	Around 800 mm/year.

* Monthly Normalized Difference Vegetation Index (NDVI). NDVI is a variable that reflects vegetation stress. Data for the period 1986–2006 [19].
