# Supplementary material for: Mexican Strains of Anaplasma marginale: A First Comparative Genomics and Phylogeographic Analysis
Source: Pathogens. 2022 Aug 2;11(8):873. doi: 10.3390/pathogens11080873 (PMC9415054; doi:10.3390/pathogens11080873)
Supplement: Supplementary file 1 [file pathogens-11-00873-s001.zip › pathogens-1809943-Table_S8.pdf]

Table S8. Evaluation of ANIm (average nucleotide identity by MUMmer) alignment coverage values of *A. marginale* genomes.

	Dawn	Florida	Gypsy Plains	Jaboticabal	MEX-01-001-01	MEX-14-010-01	MEX-15-099-01	MEX-17-017-01	MEX-30-184-02/3	MEX-30-193-01	MEX-31-096-01	Palmeira	St. Maries
Dawn	1	0.9600	0.9407	0.9708	0.9395	0.9531	0.9363	0.9376	0.9374	0.9314	0.9395	0.9718	0.9534
Florida	0.9555	1	0.9886	1.0316	1.0181	0.9937	1.0010	1.0002	1.0115	0.9937	1.0136	1.0357	1.0547
Gypsy Plains	0.9392	0.9918	1	0.9988	0.9730	0.9770	0.9671	0.9803	0.9763	0.9614	0.9751	1.0011	0.9866
Jaboticabal	0.9719	1.0378	1.0016	1	1.0204	1.0049	1.0125	1.0184	1.0128	1.0031	1.0191	1.0547	1.0438
MEX-01-001-01	0.9533	1.0380	0.9888	1.0342	1	1.0116	1.0159	1.0182	1.0296	1.0102	1.0304	1.0452	1.0374
MEX-14-010-01	0.9730	1.0192	0.9989	1.0246	1.0177	1	1.0183	1.0225	1.0174	1.0136	1.0160	1.0385	1.0210
MEX-15-099-01	0.9581	1.0292	0.9913	1.0349	1.0245	1.0208	1	1.0242	1.0207	1.0189	1.0225	1.0374	1.0180
MEX-17-017-01	0.9568	1.0256	1.0019	1.0380	1.0241	1.0222	1.0213	1	1.0256	1.0152	1.0251	1.0563	1.0286
MEX-30-184-02/3	0.9534	1.0336	0.9945	1.0289	1.0320	1.0136	1.0144	1.0221	1	1.0081	1.0432	1.0383	1.0352
MEX-30-193-01	0.9550	1.0237	0.9874	1.0274	1.0209	1.0181	1.0210	1.0201	1.0164	1	1.0166	1.0392	1.0247
MEX-31-096-01	0.9556	1.0358	0.9933	1.0353	1.0329	1.0123	1.0163	1.0217	1.0433	1.0084	1	1.0431	1.0341
Palmeira	0.9731	1.0420	1.0039	1.0548	1.0314	1.0186	1.0150	1.0364	1.0222	1.0148	1.0268	1	1.0510
St. Maries	0.9527	1.0588	0.9874	1.0417	1.0216	0.9994	0.9939	1.0072	1.0171	0.9986	1.0159	1.0488	1

NOTE: Values obtained between closely related strains are shown in colored background: Australian (black), Brazilian (orange), Mexican (blue) and North American (red).
