# Supplementary material for: Mexican Strains of Anaplasma marginale: A First Comparative Genomics and Phylogeographic Analysis
Source: Pathogens. 2022 Aug 2;11(8):873. doi: 10.3390/pathogens11080873 (PMC9415054; doi:10.3390/pathogens11080873)
Supplement: Supplementary file 1 [file pathogens-11-00873-s001.zip › pathogens-1809943-Table_S9.pdf]

Table S9. Evaluation of ANIm (average nucleotide identity by MUMmer) identity values of *A. marginale* genomes.

	Dawn	Florida	Gypsy Plains	Jaboticabal	MEX-01-001-01	MEX-14-010-01	MEX-15-099-01	MEX-17-017-01	MEX-30-184-02/3	MEX-30-193-01	MEX-31-096-01	Palmeira	St. Maries
Dawn	1	0.9856	0.9958	0.9911	0.9877	0.9906	0.9905	0.9913	0.9877	0.9907	0.9875	0.9910	0.9857
Florida	0.9856	1	0.9859	0.9857	0.9881	0.9876	0.9871	0.9875	0.9888	0.9876	0.9886	0.9855	0.9895
Gypsy Plains	0.9958	0.9859	1	0.9913	0.9879	0.9912	0.9911	0.9910	0.9879	0.9914	0.9878	0.9910	0.9864
Jaboticabal	0.9911	0.9857	0.9913	1	0.9878	0.9919	0.9912	0.9917	0.9884	0.9917	0.9885	0.9914	0.9851
MEX-01-001-01	0.9877	0.9881	0.9879	0.9878	1	0.9891	0.9893	0.9886	0.9892	0.9895	0.9889	0.9874	0.9882
MEX-14-010-01	0.9906	0.9876	0.9912	0.9919	0.9891	1	0.9914	0.9909	0.9889	0.9916	0.9890	0.9914	0.9867
MEX-15-099-01	0.9905	0.9871	0.9911	0.9912	0.9893	0.9914	1	0.9914	0.9898	0.9991	0.9895	0.9911	0.9876
MEX-17-017-01	0.9913	0.9875	0.9910	0.9917	0.9886	0.9909	0.9914	1	0.9890	0.9916	0.9889	0.9910	0.9863
MEX-30-184-02/3	0.9877	0.9888	0.9879	0.9884	0.9892	0.9889	0.9898	0.9890	1	0.9901	0.9988	0.9879	0.9887
MEX-30-193-01	0.9907	0.9876	0.9914	0.9917	0.9895	0.9916	0.9991	0.9916	0.9901	1	0.9900	0.9913	0.9872
MEX-31-096-01	0.9875	0.9886	0.9878	0.9885	0.9889	0.9890	0.9895	0.9889	0.9988	0.9900	1	0.9880	0.9885
Palmeira	0.9910	0.9855	0.9910	0.9914	0.9874	0.9914	0.9911	0.9910	0.9879	0.9913	0.9880	1	0.9849
St. Maries	0.9857	0.9895	0.9864	0.9851	0.9882	0.9867	0.9876	0.9863	0.9887	0.9872	0.9885	0.9849	1

NOTE: Values obtained between closely related strains are shown in colored background: Australian (black), Brazilian (orange), Mexican (blue) and North American (red).
